# Identifying Antifreeze Proteins Based on Key Evolutionary Information

**DOI:** 10.3389/fbioe.2020.00244

**Published:** 2020-03-26

**Authors:** Shanwen Sun, Hui Ding, Donghua Wang, Shuguang Han

**Affiliations:** ^1^Institute of Fundamental and Frontier Sciences, University of Electronic Science and Technology of China, Chengdu, China; ^2^Center for Informational Biology, University of Electronic Science and Technology of China, Chengdu, China; ^3^Department of General Surgery, Heilongjiang Province Land Reclamation Headquarters General Hospital, Harbin, China

**Keywords:** antifreeze proteins, support vector machine, evolution, machine learning, position-specific scoring matrix

## Abstract

Antifreeze proteins are important antifreeze materials that have been widely used in industry, including in cryopreservation, de-icing, and food storage applications. However, the quantity of some commercially produced antifreeze proteins is insufficient for large-scale industrial applications. Further, many antifreeze proteins have properties such as cytotoxicity, severely hindering their applications. Understanding the mechanisms underlying the protein–ice interactions and identifying novel antifreeze proteins are, therefore, urgently needed. In this study, to uncover the mechanisms underlying protein–ice interactions and provide an efficient and accurate tool for identifying antifreeze proteins, we assessed various evolutionary features based on position-specific scoring matrices (PSSMs) and evaluated their importance for discriminating of antifreeze and non-antifreeze proteins. We then parsimoniously selected seven key features with the highest importance. We found that the selected features showed opposite tendencies (regarding the conservation of certain amino acids) between antifreeze and non-antifreeze proteins. Five out of the seven features had relatively high contributions to the discrimination of antifreeze and non-antifreeze proteins, as revealed by a principal component analysis, i.e., the conservation of the replacement of Cys, Trp, and Gly in antifreeze proteins by Ala, Met, and Ala, respectively, in the related proteins, and the conservation of the replacement of Arg in non-antifreeze proteins by Ser and Arg in the related proteins. Based on the seven parsimoniously selected key features, we established a classifier using support vector machine, which outperformed the state-of-the-art tools. These results suggest that understanding evolutionary information is crucial to designing accurate automated methods for discriminating antifreeze and non-antifreeze proteins. Our classifier, therefore, is an efficient tool for annotating new proteins with antifreeze functions based on sequence information and can facilitate their application in industry.

## Introduction

Antifreeze proteins can protect cells and body fluids from freezing by hindering the nucleation, inhibiting the growth of ice crystals, and impeding the recrystallization of ice (Kandaswamy et al., 2011) and are thus important natural antifreeze materials that are widely used in food preservation (Zhan et al., 2018; Provesi et al., 2019; Song et al., 2019), medicine (Lee et al., 2012; Khan et al., 2019), and biotechnological applications (Naing and Kim, 2019). They were first found in the blood of Antarctic fishes about 50 years ago (DeVries and Wohlschlag, 1969; DeVries et al., 1970). Later studies revealed their existence in other living organisms that have to withstand sub-zero temperatures in their lifetimes, including plants (Griffith et al., 1992; Duman and Olsen, 1993), insects (Husby and Zachariassen, 1980), fungi (Duman and Olsen, 1993), and bacteria (Duman and Olsen, 1993). However, despite their superior performance at the molecular level, the quantity of many proteins that can be commercially produced is insufficient for large-scale industrial applications (Nishimiya et al., 2008). Further, some important antifreeze proteins are cytotoxic, which severely limits their potential applications (Naing and Kim, 2019). Therefore, developing tools to identify novel proteins with antifreeze functions is urgently needed.

However, in spite of similar functions among antifreeze proteins, traditional tools that search for homologous proteins based on sequence similarity, such as Basic Local Alignment Search Tool (BLAST) and Position-Specific Iterative (PSI)-BLAST, perform poorly when attempting to identify antifreeze proteins (Kandaswamy et al., 2011; Eslami et al., 2018; Nath and Subbiah, 2018), because antifreeze proteins exhibit a great diversity among species in their structures and sequence properties. For example, the ice-binding sites in fishes are moderately hydrophobic (Jia and Davies, 2002), while in plants they are mostly hydrophilic (Ramya, 2017). Distinct physicochemical and structural properties are also evident even among phylogenetically related species. Previous research on teleost fishes identified four unrelated types of antifreeze proteins, categorized by their differences in sequence and structural characteristics (Ewart et al., 1999). Type I antifreeze proteins are alanine-rich α-helical proteins; type II have C-type lectin folds of mixed α-helices and β-strands and are composed mainly of Cys, Ala, Asn, Gln, and Thr; type III are globular proteins with no particular repeated structure; type IV mainly consist of Glu and Gln and have folded α-helical bundles (Cheung et al., 2017). In insects, there are two types of antifreeze proteins that are fundamentally different in their primary, secondary, and tertiary structures despite both containing two rows of Thr residues that form β-helices (Jia and Davies, 2002). Similarly, in plants, 15 antifreeze proteins have been purified and characterized (Gupta and Deswal, 2014), and they have low homology and highly diverse properties regarding amino acid sequences (Atici and Nalbantoglu, 2003). Overall, these results suggest that antifreeze proteins may have independently evolved their ice-binding capacities (Cheung et al., 2017) and this has impeded our understanding of the relationship between sequence and function.

Despite these challenges, some researchers have attempted to build classifiers to identify antifreeze proteins based mostly on sequence-derived properties (Doxey et al., 2006; Kandaswamy et al., 2011; Zhao et al., 2012; Appels et al., 2018). For example, Doxey et al. (2006) established an algorithm to predict antifreeze proteins based on physicochemical surface features. Their method, unfortunately, is not suitable for the majority of proteins, as 3D crystallographic structures are unavailable for most proteins. Later studies on predicting antifreeze proteins used modern machine learning algorithms, which have demonstrated their ability in other protein-related research, such as identifying membrane proteins and their subcategories (Chou and Shen, 2007), predicting subcellular localization of multi-label proteins (Javed and Hayat, 2019), and classifying protein secondary structures (Ge et al., 2019). Most of these studies focused on amino acid composition-related features, and various physicochemical properties of amino acid sequences have been extensively used to identify antifreeze proteins (Kandaswamy et al., 2011; Yu and Lu, 2011; Mondal and Pai, 2014; Pratiwi et al., 2017). In contrast, despite the presumed convergent evolution of antifreeze proteins, Zhao et al. (2012) built a classifier with high performance solely based on evolutionary features derived from position-specific scoring matrices (PSSMs), suggesting that evolutionary information is also important for identifying antifreeze proteins. He et al. (2015) further compared the performances of evolutionary features with two amino acid composition metrics (i.e., amino acid composition and pseudo amino acid composition), and showed that features derived from PSSMs achieved higher performance. Similarly, Yang et al. (2015) reported that among various features pertinent to identifying antifreeze proteins, features derived from PSSMs accounted for the largest proportion, though another study showed that physicochemical properties were more important (Eslami et al., 2018). Nevertheless, these results suggest that identifying the evolutionary information underlying the differentiation between antifreeze and non-antifreeze proteins is important for increasing our understanding of protein–ice interactions.

In this study, to uncover the mechanisms of protein–ice interactions and provide an efficient and accurate automated tool for identifying antifreeze proteins, we identified key evolutionary information underlying the differentiation between antifreeze and non-antifreeze proteins. We first derived evolutionary features from PSSMs. A problem that was not resolved in most previous studies on building classifiers based on machine learning algorithms is that antifreeze proteins are rare compared to non-antifreeze proteins. This can lead the models to focusing on non-antifreeze proteins, thus impairing the training process and the assessment of model accuracy (ACC) (Yang et al., 2015). Therefore, we created a pre-processed training data set by using the Majority Weighted Minority Oversampling TEchnique (MWMOTE) to generate synthetic antifreeze proteins based on the weighted informative antifreeze proteins in the raw training data set to remedy the imbalanced training problem (Barua et al., 2014). This method uses a clustering approach to ensure that all generated antifreeze proteins are within some raw antifreeze protein clusters and has been shown to outperform several other methods (Barua et al., 2014). Thereafter, we parsimoniously selected key features to reduce redundant and noisy information based on a feature selection procedure. A classifier based on the selected key features was then trained using the support vector machine (SVM) method to discriminate antifreeze and non-antifreeze proteins.

## Materials and Methods

### Data Sets

The benchmark data sets of antifreeze and non-antifreeze proteins were obtained from Kandaswamy et al. (2011). Previously, 481 antifreeze and 9439 non-antifreeze proteins with low similarity (≤40%) were selected in the study by Kandaswamy et al. (2011), and 221 antifreeze and all the non-antifreeze protein sequences were retrieved from seed proteins in the Pfam database (Sonnhammer et al., 1997). In this study, we further removed sequences containing ambiguous residues, i.e., “X”, “B”, “U”, and “O”. In total, 479 antifreeze and 9139 non-antifreeze protein sequences were retained to derive features from PSSMs.

PSI-BLAST was used to assess the PSSM for each sequence based on sequences in the non-redundant Swiss-PROT database that share significant similarity, with three iterations and an e-value threshold of 0.0001 (Bhagwat and Aravind, 2007; Zhu et al., 2019). The raw PSSMs are *n* × 20 matrices; n rows indicate the query protein residues with n being the length of the protein sequence and 20 columns represent the 20 standard amino acids that may exist in the related protein sequences. The element in *i*th row and *j*th column assesses the frequencies of a specific amino acid (X) at position *i* in the query sequence mutating to the *j*th alternative amino acid (Z) in the related protein sequences during the evolution process. Some amino acids in the rows of each raw PSSM may appear multiple times. The rows of the same amino acids were then summed to form a 20 × 20 matrix. Thereafter, the matrix was transformed into a vector with 400 dimensions [features; for details see Zhao et al. (2012)]. Thus, each element in the vector is the occurrence of the replacement of a specific amino acid (X) in the query protein by an alternative amino acid (Z) in the related proteins, which indicates the conservation of amino acid X in each query protein. A negative (low) value of X–Z, or a positive (high) value of X–X, suggests that the mutation rate of amino acid X to Z or other amino acids is lower than expected by chance and thus X is conserved. Some sequences could not be assessed in the PSSM analysis and were, therefore, excluded. Finally, vectors based on 398 antifreeze and 7423 non-antifreeze proteins were combined into a single data set, and 80% of the antifreeze and non-antifreeze proteins were used as the training data set while the remaining 20% were used as the test data set.

The training data set was then pre-processed based on MWMOTE using the “imbalance” R package (Cordn et al., 2018) with a ratio of 0.78 being achieved between antifreeze and non-antifreeze proteins.

### Feature Selection

Features were first ranked based on the mutual information using an ensemble minimum redundancy–maximum relevance (mRMR) approach (De Jay et al., 2013; Wang et al., 2018; Yuan et al., 2018). The top ranked features were thus both the most relevant for the discrimination of antifreeze and non-antifreeze proteins and complementary to each other (Ding and Peng, 2003). Features were then added to the models sequentially starting with the one with the highest rank and the classifier was trained and evaluated based on five-fold cross-validation and the independent test data set using the SVM method (see below). To parsimoniously select key features to build the classifier to discriminate antifreeze and non-antifreeze proteins, the model preceding the one with decreased performance in the independent test data set was retained.

### Model Training and Evaluation

Support vector machine is a popular classifier which has solved several bioinformatics problems (Li et al., 2016; Chen et al., 2017; Bu et al., 2018; Zhang et al., 2018; Chao et al., 2019a, b; Sun et al., 2019; Wang et al., 2019). The “caret” R package was used to train models and tune the model hyperparameters based on SVM (Kuhn, 2008). Model performances were assessed based on ACC, sensitivity (SN), specificity (SP), and the area under the receiver operating characteristics curve (AUC) using five-fold cross-validation and the independent test data set (Tan et al., 2019). ACC is the ratio of the number of correctly discriminated proteins relative to the total number of proteins, assessing the model’s overall performance. SN is the ratio of the number of correctly discriminated antifreeze proteins relative to the number of all true antifreeze proteins. SP is the ratio of the number of correctly discriminated non-antifreeze proteins relative to the number of all true non-antifreeze proteins. In contrast, AUC considers both SN and SP, evaluating the model’s capacity to recognize antifreeze proteins among unlabeled antifreeze proteins, and non-antifreeze proteins among unlabeled non-antifreeze proteins. It is thus robust to imbalanced data. Higher AUC values indicate that a model is better at discriminating antifreeze and non-antifreeze proteins.

Additionally, to compare the performances of classifiers based on the raw data set with classifiers based on the pre-processed data set (created using MWMOTE) and the performances of classifiers based on our parsimoniously selected key features with classifiers based on all features, classifiers were also trained and evaluated using the raw data set and the pre-processed data set with all features. Additionally, principal component (PC) analysis was used to further reduce the dimensionality in all data sets and classifiers based on the first two PCs were then trained and their performances were plotted to visually illustrate the model performances. To assess the importance of each selected key feature for the first two PCs, their contributions were assessed based on the following equation:

$$\text{Contribution}=r_{i\,j}^{2}/\sum r_{i\,j}^{2}$$

where $r_{i\,j}^{2}$ is the correlation coefficient between the *i*th key feature and the *j*th PC.

## Results

### Selection of Key Features for Discriminating Antifreeze and Non-antifreeze Proteins

**FIGURE 1:**
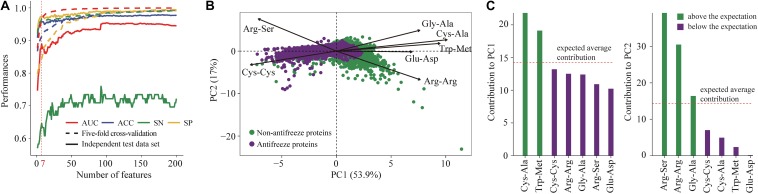
**(A)** Selection of key features derived from position-specific scoring matrices (PSSMs) for discriminating antifreeze and non-antifreeze proteins. Features were first ranked based on the mutual information using an ensemble minimum redundancy–maximum relevance (mRMR) approach. Starting with the highest ranked feature, the top 200 features were then sequentially added to the models. Model performances were assessed using five-fold cross-validation and an independent test data set based on the AUC, ACC, SN, and SP. The top seven features were parsimoniously selected to build the classifier to discriminate antifreeze and non-antifreeze proteins, and AUC, ACC, and SN then decreased in the independent test data set. **(B)** Distribution of antifreeze and non-antifreeze proteins along the first two principal components (PCs). Arrows indicate the correlations between each of the seven features and PC1 and PC2. **(C)** Contribution of each of the seven features to PC1 and PC2. Features are sorted in a descending order based on their contributions. The expected average contribution was 1/7, as there were seven features and the contribution of each feature was assumed to be uniform (Kassambara and Mundt, 2017).

Seven features derived from PSSMs were parsimoniously selected as key features for discriminating antifreeze and non-antifreeze proteins (Figure 1A). Adding more features resulted in initial reductions in performances in the independent test data set regarding AUC, ACC, and SN, although with even more features being included, the performances increased (Figure 1A). Based on the seven features, most of the proteins were correctly discriminated in the training data set, that is 96% and 97% antifreeze proteins and non-antifreeze proteins were correctly identified, respectively (Table 1). The overall ACC and AUC were 0.91 and 0.96, respectively (Table 1). In the independent test data set, a slightly lower proportion (63%) of antifreeze proteins were successfully identified, and 97% of non-antifreeze proteins were correctly predicted, which led to an increase in ACC but a decrease in AUC compared to the training data set (Table 1).

**TABLE 1:** Performances regarding discriminating antifreeze and non-antifreeze proteins based on the support vector machine (SVM) method in different data sets.

	**Features**	**Five-fold cross-validation**				**Independent test data set**			
		**AUC**	**ACC**	**SN**	**SP**	**AUC**	**ACC**	**SN**	**SP**
Raw data set	400 features	0.97	0.98	0.65	1.00	0.98	0.98	0.67	1.00
	First two PCs	0.97	0.83	0.54	1.00	0.78	0.97	0.47	1.00
Pre-processed data set^*a*^	400 features	1.00	0.99	1.00	1.00	0.96	0.98	0.70	1.00
	First two PCs	0.88	0.86	0.95	0.75	0.81	0.94	0.61	0.96
Pre-processed data set^*a*^	Seven key features	0.96	0.91	0.97	0.84	0.89	0.96	0.63	0.97
	First two PCs	0.90	0.87	0.94	0.78	0.82	0.93	0.61	0.95

*“400 features” refers to all features derived from position-specific scoring matrices (PSSMs), “first two PCs” refers to the corresponding first two principal components (PCs), and “seven key features” refers to the seven parsimoniously selected key features. ^*a*^Data set based on the Majority Weighted Minority Oversampling TEchnique (MWMOTE). AUC, area under the receiver operating characteristic curve; ACC, accuracy; SN, sensitivity, SP, specificity.*

**FIGURE 2:**
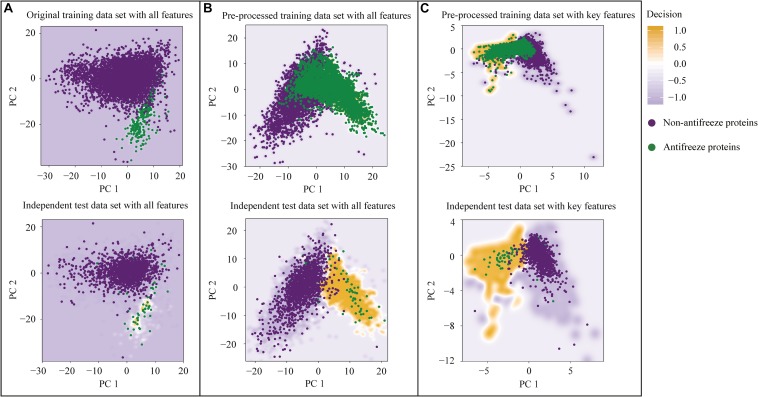
Performances of models for discriminating antifreeze and non-antifreeze proteins based on the first two principal components (PCs) derived from **(A)** all features derived from position-specific scoring matrices (PSSMs) using the raw data set, **(B)** all features derived from PSSMs using the pre-processed data set based on the Majority Weighted Minority Oversampling TEchnique (MWMOTE), and **(C)** the seven selected key features using the pre-processed data set. The upper figures are based on five-fold cross-validation and the lower figures are based on the independent test data set. See Table 1 for exact performance values. Additionally, the decision values that were used to predict the antifreeze and non-antifreeze proteins are shown.

The first two PCs derived from the seven selected key features accounted for 70% of the variation among features (Figure 1B). Along PC1, the replacements of Cys and Trp in non-antifreeze proteins by Ala and Met, respectively, in the related proteins increased in line with increasing occurrences of non-antifreeze proteins (Figures 1B,C). Similarly, along PC2, Gly and Arg in non-antifreeze proteins were more frequently replaced by Ala and Arg, respectively, in the related proteins. In contrast, there were fewer replacements of Cys, Trp, and Gly in antifreeze proteins, but more Arg was replaced by Ser (Figures 1B,C). With only the first two PCs, relatively high performances regarding discriminating antifreeze and non-antifreeze proteins were achieved (Table 1 and Figure 2C). The classifier correctly identified 94% of antifreeze proteins and 78% of non-antifreeze proteins in the training data set and 61% of antifreeze proteins and 95% of non-antifreeze proteins in the independent test data set (Table 1). The ACC and AUC were 0.87 and 0.90 in the training data set, respectively, and 0.93 and 0.82 in the independent test data set, respectively (Table 1).

### Performance of MWMOTE Method

Using the MWMOTE method to create the pre-processed data set greatly enhanced model performances. When using all features, almost every protein was correctly identified in the training data set, with SN and SP values of 1.00 and, in the independent test data set, 70% of the antifreeze proteins and 100% of the non-antifreeze proteins were correctly discriminated (Table 1 and Figure 2B). In contrast, although the classifier trained with all features and the raw data set showed overall high performances in terms of AUC, ACC, and SP, this was at the expense of correctly identifying the antifreeze proteins, i.e., a low SN (Table 1). Most of the proteins were predicted to be non-antifreeze proteins and only 65% and 67% of the antifreeze proteins were correctly recognized in the training and independent test data sets, respectively (Table 1 and Figure 2A).

## Discussion

We found that pre-processing based on the MWMOTE method improved our capacity to discriminate antifreeze and non-antifreeze proteins. Seven out of 400 features derived from PSSMs were parsimoniously selected as the key features that led to relatively high performances. There was still redundant and noisy information among these features that were minimized using a PC analysis, with a minor loss of discrimination ability. These results suggest that antifreeze and non-antifreeze proteins could be differentiated based on a few features derived from PSSMs and thus a little evolutionary information.

### Differentiation of Antifreeze and Non-antifreeze Proteins

Antifreeze proteins have been shown to have convergently evolved from different protein families (Ewart et al., 1999; Nath et al., 2013; Nath and Subbiah, 2018). Here, we found that common evolutionary relationships among antifreeze proteins may exist, i.e., Cys, Trp, and Gly are conservative and their replacements by Ala, Met, and Ala, respectively, are rare in antifreeze proteins. This result is surprising because Cys, Trp, Gly, Met, and Ala are the most hydrophobic amino acid residues (Rose et al., 1985), have been shown to have high similarities among each other in terms of hydrophobicity (Riek et al., 1995), and thus the mutation rates or replacements of Cys, Trp, and Gly by Ala, Met, and Ala, respectively, should be high (Riek et al., 1995). The conservation of Cys, Trp, and Gly in antifreeze proteins, therefore, suggests that evolutionary pressure may have existed to keep these amino acids in antifreeze proteins, and the conservation of Cys, Trp, and Gly may confer the antifreeze function on proteins, although the underlying mechanisms are still unclear. Similarly, Graham and Davies (2005) showed that, despite the surprising divergency in primary sequences, both isoforms of a highly effective antifreeze protein found in snow fleas start with Gly. Gly is thought to be very unique and highly conformationally flexible and it can occupy positions, such as tight turns, that are impossible for all other amino acids (Betts and Russell, 2003). The existence of Gly may be essential for forming various ice-binding surfaces in antifreeze proteins (Jia and Davies, 2002; Doxey et al., 2006). Moreover, the disulfide bonds formed by paired Cys residues are ubiquitous among antifreeze proteins in various taxa, including insects (Li et al., 1998; Graether et al., 2000), bacteria (Bar et al., 2006), plants (Hon et al., 1994; Bar et al., 2006), and fishes (Davies and Hew, 1990), which may enable proteins to resist destruction due to ice adsorption or denaturation stress during freezing (Li et al., 1998). Trp is an aromatic amino acid with a hydrophobic side chain, and it tends to be buried in protein hydrophobic cores, potentially forming ice-binding sites (Betts and Russell, 2003). Another possible explanation for the conservation of Cys, Trp, and Gly in antifreeze proteins is that these amino acids have higher propensities to form α-helixes (Koehl and Levitt, 1999), which is important for inhibiting the growth of ice crystals (Knight et al., 1991). In contrast to the conservation of Cys, Trp, and Gly in antifreeze proteins, Arg in antifreeze proteins was more frequently replaced by Ser and less frequently replaced by itself in the related proteins, which suggests a lack of conservation of Arg in antifreeze proteins. Similarly, Nath et al. (2013) compared the evolutionary differences between three types of antifreeze proteins in fishes and their corresponding homologous non-antifreeze proteins, and they found that Arg is commonly avoided in all types of antifreeze proteins. However, it is important to note that the PSSMs of our antifreeze proteins were based on comparing sequence similarities with related proteins but not necessarily proteins with antifreeze function. Antifreeze proteins are rare and dissimilar in their sequences, and PSI-BLAST and BLAST have difficulty using an antifreeze protein as the query sequence to search for new antifreeze proteins based on similarity (Kandaswamy et al., 2011; Eslami et al., 2018; Nath and Subbiah, 2018). Thus, some of the sequences that were used to calculate the PSSMs of our antifreeze proteins may have been non-antifreeze protein sequences. If this is the case, the high frequency of the replacement of Arg in antifreeze proteins with Ser in non-antifreeze proteins (or, in other words, the high frequency of the replacement of Ser in non-antifreeze proteins with Arg in antifreeze proteins) may indicate an important mutation contributing to antifreeze function. More stringent selection of proteins during the assessment of PSSMs could help to clarify this. Nevertheless, our results as well as the results from previous studies indicate that identifying key evolutionary information is important for understanding protein–ice interactions and for understanding the development of antifreeze proteins from pre-existing non-antifreeze proteins.

### Comparison of Our Seven Key Features With State-of-the-Art Tools for Discriminating Antifreeze and Non-antifreeze Proteins

With the advancements of genome sequencing, a large number of sequenced proteins have been accumulated and need to be functionally annotated. Many auto-annotation tools exist to identify antifreeze proteins, such as TargetFreeze (He et al., 2015), AFP_PSSM (Zhao et al., 2012), CryoProtect (Pratiwi et al., 2017), and afpCOOL (Eslami et al., 2018). However, these tools use too many features (Table 2), which may often be redundant and lead to overfitting. We found that high performances were achieved using only seven key features derived from PSSMs. Compared with other methods, our method used the smallest number of features while achieving the highest Matthews correlation coefficient (MCC), which is the correlation between predicted and true classifications and is robust to imbalanced data (Boughorbel et al., 2017), and ACC values, as well as high SN and SP (Table 2). These results indicate that our model outperforms the state-of-the-art tools and so could be more appropriate for discriminating antifreeze and non-antifreeze proteins.

**TABLE 2:** Comparison of our seven key features derived from position-specific scoring matrices (PSSMs) with existing machine learning methods for discriminating antifreeze and non-antifreeze proteins using independent test data set(s).

**Method**	**Number of features**	**ACC**	**SN**	**SP**	**MCC**
Seven key features	7	0.96	0.63	0.97	0.57
iAFP^a^	13	0.95	0.13	0.97	0.09
AFP-Pred^a^	25	0.77	0.91	0.77	0.23
AFP-PseAAC^a^	30	0.85	0.85	0.85	0.27
TargetFreeze^a^	300	0.91	0.92	0.91	0.04
CryoProtect^a^	420	0.88	0.87	0.88	0.31
AFP_PSSM^b^	400	0.93	0.76	0.93	N/A
afpCOOL^c^	641	0.96	0.72	0.98	N/A

*^*a*^Results were obtained from a study by Pratiwi et al. (2017). ^*b*^Results were obtained from a study by Zhao et al. (2012). ^*c*^Results were obtained from a study by Eslami et al. (2018). AUC, area under the receiver operating characteristic curve; ACC, accuracy; SN, sensitivity; SP, specificity; MCC, Matthews correlation coefficient. N/A: not available.*

## Conclusion

Understanding the evolution of antifreeze proteins is important for uncovering the interactions between proteins and ice, and, more broadly, the adaptation of organisms to their environments. We found that the conservation of several key amino acids showed opposite tendencies in antifreeze and non-antifreeze proteins, suggesting that there has been strong selection pressure related to these amino acids leading to the differentiation between antifreeze and non-antifreeze proteins regarding their ice-binding capacities. Moreover, we showed that evolutionary information is crucial for designing accurate automated tools for discriminating antifreeze and non-antifreeze proteins. Therefore, our model, which is based on seven key features derived from PSSMs and outperforms the state-of-the-art tools, is an efficient and crucial tool to help to identify new antifreeze proteins and facilitate their use.

## Data Availability Statement

Publicly available datasets were analyzed in this study. This data can be found in Kandaswamy et al. (2011).

## Author Contributions

SS, HD, DW, and SH: conceptualization. SS: formal analysis and writing and preparation of the original draft. SS, HD, DW, and SH: writing-review and editing. All authors have read and agreed to the published version of the manuscript.

## Conflict of Interest

The authors declare that the research was conducted in the absence of any commercial or financial relationships that could be construed as a potential conflict of interest.
